# Neonatal vitamin A supplementation and immune responses to oral polio vaccine in Zimbabwean infants

**DOI:** 10.1093/trstmh/try126

**Published:** 2018-12-20

**Authors:** James A Church, Sandra Rukobo, Margaret Govha, Marya P Carmolli, Sean A Diehl, Bernard Chasekwa, Robert Ntozini, Kuda Mutasa, Jean H Humphrey, Beth D Kirkpatrick, Andrew J Prendergast

**Affiliations:** 1 Zvitambo Institute for Maternal and Child Health Research, 16 Lauchlan Avenue, Harare, Zimbabwe; 2 Centre for Genomics & Child Health, Blizard Institute, Queen Mary University of London, Newark Street, London, UK; 3 Vaccine Testing Center, Larner College of Medicine, University of Vemont, Burlington, VT, USA; 4 Department of International Health, Johns Hopkins Bloomberg School of Public Health, Baltimore, MD, USA

**Keywords:** Africa, infants, OPV, oral vaccine, poliovirus, vitamin A

## Abstract

**Background:**

Micronutrient deficiencies may contribute to reduced oral vaccine immunogenicity in developing countries. We hypothesised that neonatal vitamin A supplementation (NVAS) would improve oral vaccine responses.

**Methods:**

We performed a cross-sectional study of infants recruited at birth to the Zimbabwe Vitamin A for Mothers and Babies (ZVITAMBO) trial, a randomised controlled trial of single, high-dose NVAS vs placebo conducted in Zimbabwe between 1997–2001. We measured poliovirus-specific IgA to type 1–3 polio strains by semiquantitative capture ELISA in cryopreserved plasma samples collected at 6 months of age.

**Results:**

A total of 181 infants fulfilled inclusion criteria, of whom 80 were randomised to NVAS and 101 to placebo. There were no significant differences in baseline characteristics between groups. At 6 months of age, median (IQR) vaccine titres for infants randomised to NVAS vs placebo were 932 (421–3001) vs 1774 (711–5431) for Sabin-1 (p=0.04); 1361 (705–3402) vs 2309 (1081–4283) for Sabin-2 (p=0.15); and 1584 (796–4216) vs 2260 (996–5723) for Sabin-3 (p=0.14), respectively. After adjusting for breast feeding status, birth weight, season and infant sex in a linear regression model, there was only weak evidence of difference in log mean titres between vitamin A and placebo groups for Sabin-1 (p=0.08) and no evidence of difference in log mean titres for Sabin-2 and Sabin-3.

**Conclusions:**

NVAS did not augment oral polio vaccine responses in Zimbabwean infants. Further research is required to understand the impact of NVAS on responses to other oral vaccines.

The trial is registered with clinicaltrials.gov identifier: NCT00198718.

## Background

Oral vaccines consistently underperform when given to children in developing countries, substantially reducing their public health benefit.^1^ Impaired immunogenicity of oral polio vaccine (OPV) has hampered the global eradication of polio^2^ while oral rotavirus vaccine efficacy against severe rotavirus gastroenteritis is as low as 39% in sub-Saharan Africa.^3^ Interventions to improve oral vaccine efficacy have been impeded by an incomplete understanding of the biological mechanisms underpinning poor vaccine performance.^1^ Several factors have been postulated to reduce oral vaccine immunogenicity in developing countries including genetic determinants, concurrent enteric infections, environmental enteric dysfunction, interference from breast milk antibodies, and deficiency of micronutrients such as zinc and vitamin A^4–8^; however, their relative contributions remain unclear.^1^

Vitamin A deficiency is highly prevalent in regions where oral vaccines underperform and is a major contributor to infection, blindness and mortality.^9^ Periodic vitamin A supplementation (VAS) is therefore recommended by WHO for children from 6–59 months in developing countries and has been a key strategy to reduce morbidity and mortality.^10^ The role of neonatal vitamin A supplementation (NVAS), however, has been more uncertain.^11^ In view of the benefits of VAS in older children together with high neonatal mortality and vitamin A deficiency in women of reproductive age in developing countries, WHO recently evaluated NVAS in a series of trials in Ghana, Tanzania and India.^12–14^ NVAS did not reduce infant morbidity or mortality in sub-Saharan Africa and in India there was an initial survival benefit at 6 months of age, which was no longer seen at 12 months.^14^

Vitamin A is an immunomodulator and integral to healthy mucosal immune responses. Animal studies have shown that vitamin A deficiency impairs vaccine-elicited gastrointestinal immunity^15^ and derivatives of vitamin A have adjuvant potential when given with vaccines.^16^ It is therefore biologically plausible that VAS may improve responses to early childhood vaccines. However, the interaction between VAS and responses to vaccines at a community level is poorly understood. A number of studies have looked at associations between VAS and immune responses to both parenteral and oral vaccines, mostly in older children but also among children from 6 weeks of age, with mixed findings (reviewed in^17^). To our knowledge, the impact of NVAS on oral vaccine responses has not been described. The purpose of this study was to examine whether vitamin A given to infants soon after delivery could boost subsequent responses to oral vaccines. We compared immune responses to OPV in a well-characterised cohort of Zimbabwean infants randomised to receive high-dose NVAS or placebo at birth.

## Methods

### Study design

This cross-sectional study used archived samples from the Zimbabwe Vitamin A for Mothers and Babies (ZVITAMBO) trial (clinicaltrials.gov identifier NCT00198718). ZVITAMBO was a 2×2 factorial randomised trial carried out between 1997 and 2001 to evaluate the effects of a single high-dose of vitamin A or placebo given to mothers and their infants during the immediate postpartum period, on several infant health outcomes.^18–21^ Briefly, 14 110 mother-infant pairs were enrolled within 96 hours of delivery from clinics in Harare, Zimbabwe and randomly assigned to one of four treatment groups in a factorial design: Aa, Ap, Pa, and PP, where ‘A’ denoted maternal vitamin A supplementation (400 000 IU), ‘P’ was maternal placebo, ‘a’ was infant vitamin A supplementation (50 000 IU) and ‘p’ was infant placebo. Mother-infant pairs were eligible if neither had an acutely life-threatening condition and the infant was a singleton with birth weight >1500 g. Three-quarters of the mother-infant pairs received their treatment dose within 24 hours, and 94% received it within 48 hours of delivery.

Follow-up was conducted at six weeks, three months and then every three months to 12–24 months of age. Anthropometry was performed at each visit, using methods previously described,^22^ with Z-scores calculated based on the WHO 2010 reference standards using WHO Anthro version 3.0.1 (http://www.who.int/childgrowth/en). All mothers were encouraged to exclusively breast feed their infants for six months. Data on feeding practices obtained at six weeks, three months and six months was used to categorise infants as exclusively, predominantly or mixed breast fed.^23^

At the time, there were no national VAS programmes for postpartum women or neonates in Zimbabwe and VAS among older children was not initiated until the end of the trial. Based on a random subsample of 375 HIV-negative mothers who had measurements at delivery, 37.1% across both groups had serum retinol levels <1.05 *μ*mol/litre.^20^ However, at six weeks postpartum, serum retinol levels increased to within normal limits in most women, reflecting either a normalisation of pregnancy-associated haemodilution or inflammation in the postpartum period.

### Biological specimen collection

Blood was collected from all enrolled mothers and infants at baseline and from a representative subsample (52% of total) of mother-infant pairs at all follow-up visits. Samples were centrifuged and plasma removed within two hours of blood collection. Samples were stored in -80°C freezers with automatic generator backup. Mothers underwent HIV testing at baseline using two parallel ELISA assays. Women testing HIV-negative were re-tested at every visit to detect HIV seroconversion. Children were classified as HIV-unexposed if the mother tested HIV-negative at baseline and did not seroconvert during follow-up.

### Selection of study infants

For the current study, we retrieved samples for all infants fulfilling the following criteria: (1) the mother received placebo in the original trial (‘P’); (2) infants were HIV-unexposed (i.e., born to mothers who remained HIV-negative until 12 months postpartum); (3) infants were followed to 6 months of age with available feeding and anthropometry data; (4) a sufficient sample of cryopreserved plasma was available for laboratory assays at 6 months of age. We did not include infants born to mothers who received vitamin A because our hypothesis was specifically that infant vitamin A supplementation would boost OPV responses.

### Oral polio vaccination

Infants followed the routine Expanded Programme of Immunisation (EPI) schedule in Zimbabwe at the time, which included trivalent OPV at 3, 4 and 5 months of age. Infants did not receive OPV at birth. Specific vaccination data were not collected as part of the trial, but immunisation coverage of the third dose of polio vaccine (given between 3 and 4 months of age) was 70–81% at that time in Zimbabwe (1997–2000).^24^

### Antigen-capture ELISA for detection of poliovirus IgA

Although neutralising antibody titre is the best correlate of protection for OPV,^25,26^ measurements of circulating (serum) IgA responses to poliovirus are useful in the detection and control of poliovirus infection.^27–32^ In all six-month plasma samples (i.e., one month after receipt of final OPV), we measured poliovirus-specific IgA to type 1–3 polio strains, based on a previously described capture ELISA technique,^33^ using Sabin antigen cultured from Hep2C cells at the University of Vermont. Pooled serum from poliovirus-vaccinated healthy donor volunteers was used as a positive control; each plate also included a column of blank wells with no test sample as a negative control. Using threefold serial dilutions of patient samples run out in a single column allowed for a semiquantitative measure of poliovirus-specific IgA, an adaptation of the original method. Endpoint titres were calculated by subtracting the plate background (i.e., the average optical density [OD] of the negative control wells), then identifying the dilution of the final well in each sample column with an OD >0.07 (a 95% confidence value cut-off used to distinguish negative from positive absorbance values and calculated according to the original method).^33^ If an endpoint titre could not be derived at the first attempt (e.g., bottom well OD ≥0.08), the assay was repeated using a 10-fold higher or lower concentration of patient sample where sufficient serum volume was available. If an endpoint titre could not be derived using the new sample concentration, the lowest or highest dilution factor was taken as the final endpoint. Extreme low and high endpoint titres were subsequently truncated and assigned a value equivalent to the 5th and 95th centile within the data set, respectively.

The intra- and inter-plate coefficients of variation for Sabin-1, -2 and -3 strains, derived from the mean ODs and SDs from seven successive preliminary experiments run prior to including study samples, were 8.2%, 4.0% and 4.8% and 20.1%, 17.7% and 16.8%, respectively. As a means of continued quality assurance, the endpoint titre of the positive control sample in all subsequent assays had to fall within an acceptable range for the experiment to be deemed valid. The range was predetermined based on the upper and lower limit ODs in the seven preliminary experiments. Laboratory scientists were blinded to infant vitamin A status when conducting the assays.

### Statistical analysis

Baseline characteristics were compared between groups using Chi-squared test for categorical variables, and for continuous variables two-sample t-test or Wilcoxon-Mann-Whitney for normally distributed and skewed data distributions, respectively. All vaccine titres were positively skewed and were log_10_ transformed to normality. A linear regression model was fitted to calculate adjusted differences between log_10_ mean vaccine titres between groups, using breast feeding, birth weight and infant sex as preselected covariates based on biological plausibility.^4,34,35^ All statistical analyses were performed using STATA 14 (StataCorp LP, College Station, TX, USA) and Prism v6 (GraphPad Software Inc., CA, USA).

## Results

A total of 181 children fulfilled the inclusion criteria for this study, of whom 80 were randomised to NVAS and 101 to placebo. Baseline characteristics of infants and their mothers are shown in Table 1. There were no significant differences in maternal or infant variables between groups and baseline characteristics for this study were comparable to those of the larger population in the original trial.^21^ All mothers were still breast feeding at six months postpartum (Table 1), but most infants were mixed fed; exclusive breast feeding rates were low overall (5.0% in both groups).

**Table 1.** Baseline characteristics of infants and mothers

	Vitamin A group	Placebo group	p value
	N=80	N=101	
**Infant characteristics**			
Male sex, % (n)	47.5 (38)	52.5 (53)	0.51
Gestational age, weeks; mean (SD)	39.3 (1.5)	39.2 (2.1)	0.72
Preterm (<37 weeks), % (n)	10.1 (8) [79]	11.0 (11) [100]	1.00
Birth weight, kg; mean (SD)	3.01 (0.45)	3.01 (0.44)	1.00
Low birth weight (<2500 g), % (n)	12.5 (10)	13.9 (14)	0.83
Birth length, cm; mean (SD)	47.7 (2.5)	47.8 (2.4)	0.79
Birth head circumference, cm; mean (SD)	34.3 (1.5)	34.1 (2.1)	0.47
Normal vaginal delivery, % (n)	85.0 (68)	86.7 (85) [98]	0.74
Exclusive breast feeding*, % (n)	5.0 (4)	5.0 (5)	0.99
Predominant breast feeding*, % (n)	27.5 (22)	23.8 (24)	0.57
Mixed feeding*, % (n)	67.5 (54)	71.3 (72)	0.58
Maternal characteristics**			
Age, years; mean (SD)	25.6 (6.0)	24.6 (5.7)	0.25
Married or stable union, % (n)	90.0 (72)	95.0 (96)	0.19
Education, years; median (IQR)	11 (9,11)	11 (9,11)	0.67
Parity, median (IQR)	2 (1,3)	2 (1,3)	0.53
Maternal MUAC, cm; mean (SD)	26.0 (3.0)	26.4 (3.1)	0.38
Unemployed, % (n)	81.3 (65)	86.1 (87)	0.79
Household income, US$ per month; median (IQR)	79.3 (60, 121)	76.5 (52, 130)	0.54

[x] refers to total number if data missing.

* Breast feeding status assessed at six months postpartum.

** All mothers received placebo as per selection criteria for this study.

p values are shown since this is a subgroup of infants who were randomised to vitamin A or placebo in the original ZVITAMBO trial.

**Figure 1.**
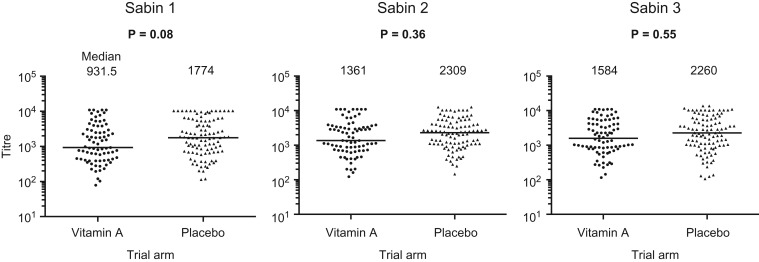
Immune responses to OPV: plasma polio-specific IgA endpoint titres (Sabin-1, -2 and -3) at 6 months of age in infants randomised to neonatal vitamin A or placebo. P-values were derived from a linear regression model, adjusting for breast feeding status, birth weight, season and infant sex. Very few infants were omitted from the multivariable analysis. In each case the missing covariate data was sex and birth weight. For Sabin-1, 5/181; for Sabin-2, 3/181; for Sabin-3, 1/181 infants.

At 6 months of age (one month post-immunisation), median (IQR) vaccine endpoint titres for infants randomised to NVAS vs placebo were 932 (421–3001) vs 1774 (711–5431) for Sabin-1 (p=0.04); 1361 (705–3402) vs 2309 (1081–4283) for Sabin-2 (p=0.15); and 1584 (796–4216) vs 2260 (996–5723) for Sabin-3 (p=0.14), respectively. After adjusting for breast feeding status, birth weight, season and infant sex in a linear regression model, there was only weak evidence of difference in log mean endpoint titres between vitamin A and placebo groups for Sabin-1 (coefficient −0.15, 95% CI −0.31, 0.02; p=0.08) and no evidence of difference in log mean titres for Sabin-2 (−0.07, 95% CI −0.23, 0.08; p=0.36) or Sabin-3 (−0.05, 95% CI −0.21, 0.11; p=0.55) (Figure 1). We also tested for an interaction between vitamin A exposure and infant sex on OPV response, but found no interaction for each Sabin strain with p*-*values of 0.43, 0.34 and 0.39, respectively. Similarly, there was no evidence that low birth weight, which may predispose to vitamin A deficiency, modified the effect of vitamin A exposure on OPV response (data not shown).

## Discussion

In this study, we show that high-dose vitamin A administered to newborn infants within 96 hours of delivery does not improve OPV immunogenicity at 6 months of age compared to placebo. We are not aware of any studies that have published data on the impact of NVAS on oral vaccine responses previously; however, our findings are consistent with three randomised controlled trials in Bangladesh,^36^ Indonesia^37^ and Ghana^38^ where vitamin A was given concurrently with doses of OPV at 6, 10 and 14 weeks of age. The Ghanaian study, which examined the independent and combined effects of maternal and infant VAS among 1085 pairs, showed no differences in antibody titres to either OPV or tetanus in any of the groups.^38^ Only one study to our knowledge, conducted in India, found that children supplemented with vitamin A at 6, 10 and 14 weeks of age, had significantly improved responses after trivalent OPV, and this was restricted to Sabin-1 responses.^39^ In contrast, we saw a slight trend for a reduction in antibody titre for Sabin-1, but this was not significant in the multivariable analysis.

The impact of NVAS on mortality has produced conflicting results, with some trials in south Asia showing benefit^14,40^ whereas those in sub-Saharan Africa show a null effect or even harm.^12,13^ A recent Cochrane review concluded that overall NVAS confers no significant reduction in mortality at 6 or 12 months of age. However, the meta-analysis combines estimates from all African and Asian trials and the resulting global conclusion may not be appropriate given such a marked variability in effect by region. Meanwhile, a policy recommendation from WHO around the use of NVAS has stalled.^41^ Drawing firm conclusions on the impact of vitamin A on vaccine responses is similarly hampered by differences in nutritional landscapes, phenotypes and study designs; in particular, vitamin A status is often not measured but is likely to vary between settings. Although WHO categorised Zimbabwe as ‘high-risk’ for vitamin A deficiency at the time of the original ZVITAMBO trial,^42^ the prevalence of vitamin A deficiency among this subset of newborns may have been low. All infants were breast fed and vitamin A deficiency was rare among HIV-negative mothers recruited to the trial (only 9% had serum retinol levels <0.7 μmol/litre).^19^ Unfortunately, we did not have serum retinol levels in these infants to provide a precise measure of vitamin A status. It is plausible that VAS has a boosting effect on OPV responses in vitamin A-deficient infants (as described in the India study^39^), which we were unable to detect here. Maternal vitamin A status can also modify infant outcomes.^43^ We were therefore careful in our study to include only those infants born to mothers randomised to the placebo arm.

This study had several strengths. It took advantage of a repository of samples from a well-characterised birth cohort, and randomised groups in this substudy had similar baseline characteristics. Moreover, this was a unique opportunity to evaluate the impact of NVAS on oral vaccine responses. There were a few important limitations to our study. First, immunisation records were not collected in the original ZVITAMBO trial and so we cannot be sure how many doses of OPV each infant received. However, vaccine uptake was high during this period in Zimbabwe and we cannot envisage a plausible reason for differences in OPV dosing between vitamin A and placebo groups. Second, we did not have access to the gold-standard serum neutralising assay to measure response to OPV, because it was unavailable in our setting. Therefore, while the ELISA we used to detect polio-specific IgA enables a semiquantitative measure of immunogenicity,^33^ we are unable to draw conclusions about protection from OPV. Third, our sample size was limited by the number of cryopreserved specimens with sufficient volume still remaining from the original trial. We may have therefore been underpowered to detect differences between groups. Finally, without available samples to measure baseline IgA titres, we cannot rule out a difference between groups in antibody rise between pre- and postvaccination.

## Conclusions

Although an effect of vitamin A on oral vaccine responses is biologically plausible, NVAS at birth did not augment OPV responses in this population of Zimbabwean infants. However, in regions such as south Asia where the use of NVAS may be scaled up due to mortality benefits, further studies are warranted to better understand the impact of vitamin A on oral vaccine responses, particularly where new oral vaccinations such as rotavirus are being introduced.

## Supplementary data

Supplementary data are available at Transactions online (http://trstmh.oxfordjournals.org/).

## Supplementary Material

Supplementary Data
