# Supplementary material for: Neonatal vitamin A supplementation and immune responses to oral polio vaccine in Zimbabwean infants
Source: Trans R Soc Trop Med Hyg. 2018 Dec 20;113(3):110–5. doi: 10.1093/trstmh/try126 (PMC6391935; doi:10.1093/trstmh/try126)
Supplement: Supplementary Data [file try126_180717_consort_flow_diagram.pptx]

## Slide 1
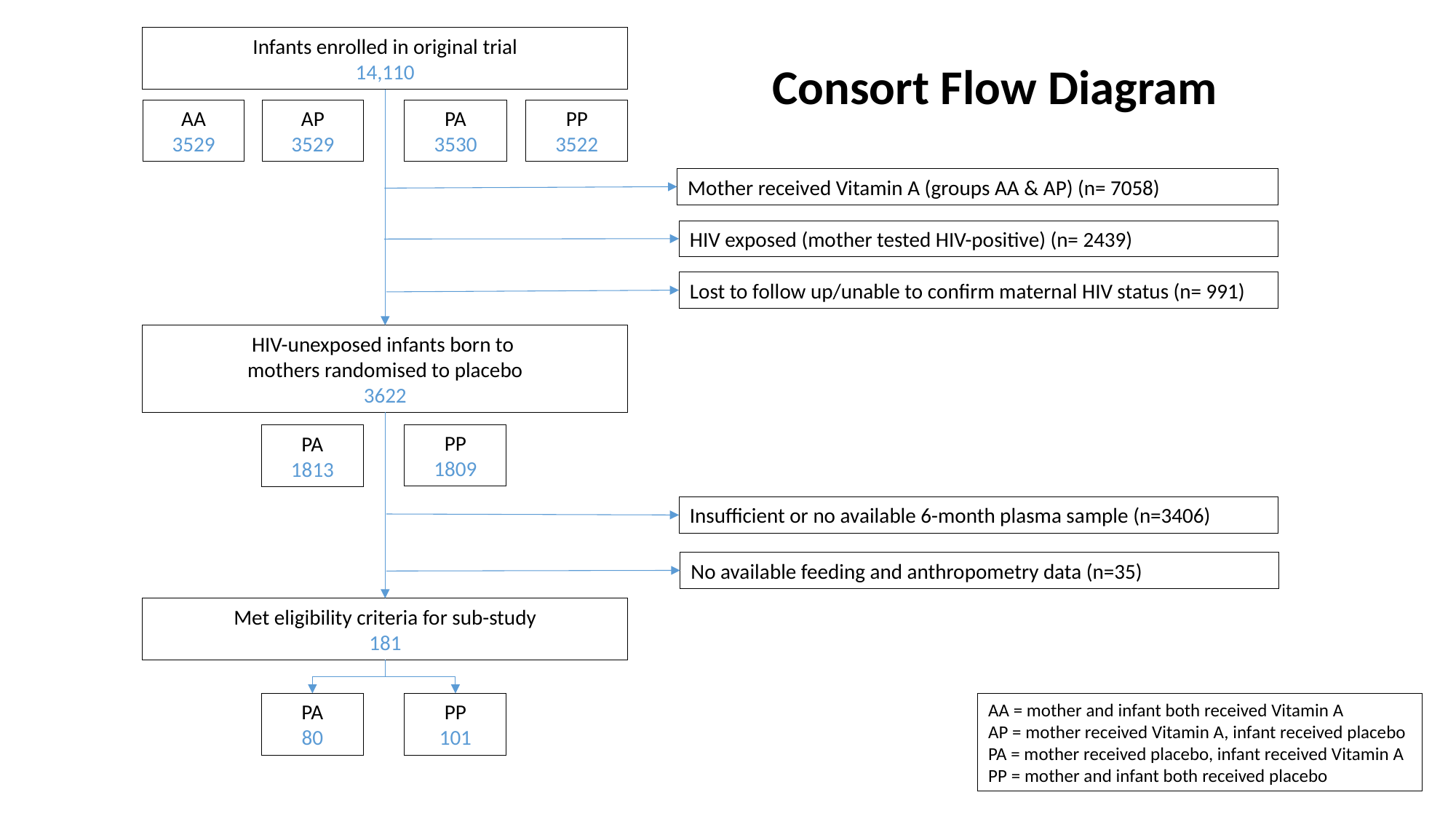

Infants enrolled in original trial
14,110
Consort Flow Diagram
AA
3529
AP
3529
PA
3530
PP
3522
Mother received Vitamin A (groups AA & AP) (n= 7058)
HIV exposed (mother tested HIV-positive) (n= 2439)
Lost to follow up/unable to confirm maternal HIV status (n= 991)
HIV-unexposed infants born to
mothers randomised to placebo
3622
PP
1809
PA
1813
Insufficient or no available 6-month plasma sample (n=3406)
No available feeding and anthropometry data (n=35)
Met eligibility criteria for sub-study
181
AA = mother and infant both received Vitamin A
AP = mother received Vitamin A, infant received placebo
PA = mother received placebo, infant received Vitamin A
PP = mother and infant both received placebo
PA
80
PP
101
